# Are special read alignment strategies necessary and cost-effective when handling sequencing reads from patient-derived tumor xenografts?

**DOI:** 10.1186/1471-2164-15-1172

**Published:** 2014-12-23

**Authors:** Kai-Yuen Tso, Sau Dan Lee, Kwok-Wai Lo, Kevin Y Yip

**Affiliations:** Department of Computer Science and Engineering, The Chinese University of Hong Kong, Shatin, New Territories, Hong Kong; Department of Anatomical and Cellular Pathology, The Chinese University of Hong Kong, Shatin, New Territories, Hong Kong; Hong Kong Bioinformatics Centre, The Chinese University of Hong Kong, Shatin, New Territories, Hong Kong; CUHK-BGI Innovation Institute of Trans-omics, The Chinese University of Hong Kong, Shatin, New Territories, Hong Kong

**Keywords:** Xenografts, Nasopharyngeal carcinoma, Contamination, High-throughput sequencing

## Abstract

**Background:**

Patient-derived tumor xenografts in mice are widely used in cancer research and have become important in developing personalized therapies. When these xenografts are subject to DNA sequencing, the samples could contain various amounts of mouse DNA. It has been unclear how the mouse reads would affect data analyses. We conducted comprehensive simulations to compare three alignment strategies at different mutation rates, read lengths, sequencing error rates, human-mouse mixing ratios and sequenced regions. We also sequenced a nasopharyngeal carcinoma xenograft and a cell line to test how the strategies work on real data.

**Results:**

We found the "filtering" and "combined reference" strategies performed better than aligning reads directly to human reference in terms of alignment and variant calling accuracies. The combined reference strategy was particularly good at reducing false negative variants calls without significantly increasing the false positive rate. In some scenarios the performance gain of these two special handling strategies was too small for special handling to be cost-effective, but it was found crucial when false non-synonymous SNVs should be minimized, especially in exome sequencing.

**Conclusions:**

Our study systematically analyzes the effects of mouse contamination in the sequencing data of human-in-mouse xenografts. Our findings provide information for designing data analysis pipelines for these data.

**Electronic supplementary material:**

The online version of this article (doi:10.1186/1471-2164-15-1172) contains supplementary material, which is available to authorized users.

## Background

Xenotransplantation is the transplantation of living cells, tissues or organs from one species to another. Patient-derived tumor xenografts (PDX) in mice have been used as an important model in cancer research, where human cancer cells or tumor tissues are transplanted to immunodeficient mice to study the molecular characteristics of the tumors, identify factors involved in malignant transformation, invasion and metastasis, and predict the efficacy and toxicities of cancer chemotherapeutic agents [1–5]. Recently, there has been a new trend of using patient-derived tumor xenografts to develop personalized medicine and anticancer therapies tailored for the patient [6]. This use of xenografts is expected to turn this traditional research tool into large-scale clinical use.

During the expansion of a xenograft in mice, human stromal cells are largely replaced by mouse stromal cells. When cell samples from a xenograft are extracted for DNA sequencing, the samples could contain various amounts of mouse DNA. In a study of 93 xenografts for studying human pancreatic cancer, the estimated fraction of contaminating mouse DNA ranged from 17% to as high as 73%, with an average of 47% [7]. Due to the high similarity between the human and mouse genomes, sequencing reads originating from mouse DNA could affect the results of various kinds of analyses. For example, if a contaminating mouse read is aligned to the human reference with a mismatch, the mismatch may be wrongly treated as a single nucleotide variant (SNV) in the cancer cells. Mixed DNA could also affect the analysis of copy number variations [8, 9].

In the literature, there is limited previous work that discusses how human sequencing reads with mouse contamination should be handled. Conway et al. proposed two computational methods for classifying human and mouse reads [10], which were tested on RNA-seq data from human, mouse, and human-in-mouse xenografts. These methods, collectively called Xenome, align each read to the human and mouse references independently, and report whether it aligns to the human reference only, the mouse reference only, both, or neither (as well as some ambiguous cases in the k-mer version of the method). In another study, Valdes et al. analyzed sequences from RNA-seq experiments that cross-aligned between the mouse and human genomes [11]. Some sequencing centers have established pipelines for mapping sequencing reads from xenografts to both human and mouse genomes simultaneously and selecting the reads that map better to the human reference [12]. An implicit assumption behind these methods is that contaminating mouse sequences need to be specially handled in order to have accurate downstream analysis results. In contrast, there were also studies that decided not to explicitly handle contaminating mouse data and assumed they would not significantly affect analysis results as the estimated percentage of mouse DNA was low [13].

Is it necessary and cost-effective to perform special handling of human sequencing reads contaminated with mouse data? Since special handling is not part of the standard data processing pipelines, it may incur extra labor cost. In addition, as to be explained below, some special handling methods require more processing time and/or memory space. It is not very obvious whether such extra cost is worth paying for, due to the following two reasons.

On the one hand, while the human and mouse genomes are highly similar as compared to other more evolutionarily distant species, at the DNA level their coding sequence similarity is only 85% on average [14]. Even for a "short" read with 50-100 nucleotides, it is not immediately clear what fraction of reads originated from mouse would be aligned to the human reference.

On the other hand, if human and mouse genomes can indeed produce very similar reads, it is then unclear if the benefits of special handling, such as filtering out some mouse reads and thus reducing false calling of SNVs, would be offset by undesirable side-effects, such as accidental filtering of human reads that causes some legitimate SNVs to be missed.

To answer these questions, we have carried out a comprehensive simulation study with sequencing reads generated from the human and mouse genomes. To take into account various scenarios that could happen in real settings, we have considered a large combination of values of different simulation parameters, including:

-  Rate of mutation between the reference genome sequences and the mutated sequences we used for generating short reads
-  Rate of sequencing errors
-  Length of sequencing reads
-  Mixing proportion of human and mouse reads
- Sequencing reads generated from exonic regions only or both exonic and non-exonic regions

We compared three different strategies for handling the mixed human and mouse reads, with their effectiveness evaluated by the resulting accuracies of both sequence alignment and genetic variant identification. The use of simulated data allowed us to compute the accuracy based on the actual origin and DNA sequence of each sequencing read.

To further test how well the three strategies work on real data, we performed deep sequencing of a nasopharyngeal carcinoma (NPC) PDX implanted in nude mice and an NPC cell line that should not have mouse contamination as control. We then applied the three strategies on these two sets of data and compared their results.

## Methods

### Three computational strategies for handling sequencing reads with contaminated data

**Direct mapping.** The first strategy we considered is to map all sequencing reads directly to the human reference genome. There are three situations in which this strategy would be applied. First, if the contamination rate is known or believed to be low, or most mouse reads are unlikely to align well to the human genome due to long read length, one may expect the contamination has limited effects on the data analysis and apply this strategy. Second, some sequencing services may include a standard data processing pipeline for mapping reads to the human reference, but do not offer non-standard handling of potential mouse reads, or not without a cost. Finally, one may be unaware of the presence of mouse contamination in the sample and thus does not consider any special handling of the sequencing reads.

False positives are the major potential problem of this strategy. Some mouse reads could be falsely aligned to the human reference, and the differences between these reads and the aligned regions could be wrongly interpreted as genetic variants. Although more unlikely, false negatives are also possible as the calling of genetic variants could be sensitive to the portion of reads supporting each allele. For example, in a normal sample without aneuploidy, heterozygous SNVs would be easier to detect if the number of reads supporting each allele is close to half. In the rare but possible event that mouse reads can align to the locus well and support one of the two alleles, the other allele could be missed due to a reduced detection score. There are variant callers specifically designed for tumor samples that can identify somatic variants by comparing read counts in a tumor sample and a corresponding control. While these callers can detect variants with reads supporting each allele substantially deviating from the normal case, they can also be affected by false positive and false negative reads caused by mouse contamination.

**Filtering.** The second strategy is to filter out mouse-like reads before mapping to the human reference, by first aligning all sequencing reads to the mouse reference and discarding those with a high alignment score. The remaining reads are then collected and aligned to the human reference. The resulting set of aligned reads is equivalent to those that can be aligned to the human reference only by Xenome, although in our approach not all reads need to be aligned to both references in order to identify this subset. The filtering strategy is used when one wants to minimize the amount of mouse reads falsely aligned to the human reference and the incorrectly identified genetic variants thereof. For instance, it could be the strategy of choice when one can only experimentally validate a small number of identified genetic variants, and would thus want to minimize false positives among them.

This strategy could wrongly filter out human reads that are highly similar to some regions in the mouse genome, leading to false negatives.

If the contamination rate is not high, most reads would be aligned twice, first to the mouse genome (unsuccessfully) and then to the human genome. The computational time required would therefore double that of the direct mapping strategy. Modifications to sequence alignment pipelines are also needed to extract the unaligned reads after mapping to the mouse reference and remap them to the human reference.

**Mapping to combined reference.** The third strategy is to combine the human and mouse reference genomes into an artificial genome, and align all sequencing reads to it. This strategy mainly differs from the filtering strategy in how it deals with ambiguous reads that have high sequence similarity to both human and mouse genomes. Whereas in the filtering strategy ambiguous reads are filtered in the first step, in the combined reference strategy each read is mapped to the most similar region in the two genomes as long as it is the only best match in the two genomes. The resulting set of reads cannot be directly obtained from Xenome, because the Xenome method does not further suggest how the reads that align to both references should be handled, but here we also report to which reference each of them aligns better.

This strategy is expected to give a lower false negative alignment rate than the filtering strategy, because some ambiguous reads originated from human data can be successfully mapped to the human reference. The false positive rate is expected to be intermediate between the other two strategies.

One disadvantage of this strategy is the need to construct a large artificial genome of about 6 billion nucleotides. The corresponding indexing structure could take up 6G bytes of main memory, which exceeds the capacity of computing machines that run a 32-bit operating system (with a memory limit of 4 G bytes per process). There are also implementations of alignment tools, such as an older version of Bowtie2 [15], that cannot handle such a huge reference genome regardless of the operating system on which it is run.

### Simulation parameter profiles

Our comprehensive simulation study involved different combinations of values of five simulation parameters. Three of these parameters with binary values were first used to construct 8 setting profiles (Table 1).

**Table 1:** **The different profiles of parameter settings considered in our study**

Profile	Mutation rate	Read length	Error rate
mL.len050.eL	0	50	0.0001
mL.len050.eH	0	50	0.01
mL.len100.eL	0	100	0.0001
mL.len100.eH	0	100	0.01
mH.len050.eL	0.01	50	0.0001
mH.len050.eH	0.01	50	0.01
mH.len100.eL	0.01	100	0.0001
mH.len100.eH	0.01	100	0.01

Mutation rate is the probability for any base to be altered by an SNV or indel from the reference genome, before the resulting mutated genome was used to generate artificial sequencing reads. It was applied to both the human and mouse reads we generated. The purpose of this parameter was to investigate how the differences between a (human or mouse) genome and the corresponding reference genome could affect read alignment results. A high mutation rate would increase the chance for a read generated from the human genome to be better aligned to the mouse reference than the human reference, and vice versa. We considered two mutation rates in our simulation study, namely zero mutation rate for studying the ideal case, and a high mutation rate of 1% with SNV to indel ratio of 9:1, for studying the more difficult situations.

Read length is the number of base pairs (bp) on each simulated sequencing read. A smaller read length would increase the chance for a read to be aligned to a wrong reference or non-uniquely aligned to two loci in a reference. Reads failing to be unambiguously aligned to a unique location would be marked as having low alignment quality, which would in turn affect the ability to identify any genetic variants it contains. We considered two read lengths in our study, namely 100 bp for typical reads based on current standards, and 50 bp for relatively short reads.

Error rate is the probability for a base on a simulated read to be wrongly sequenced. Both simulated mutation and sequencing error can produce reads that are different from the original reference sequence, but only the latter has a corresponding reduced quality score, which can be utilized by read alignment and variant calling algorithms. We tried two different error rates in our study, namely a low error rate of 0.01%, and a high error rate of 1%.

We considered all value combinations of these three parameters to form 8 setting profiles (Table 1). The profiles are named systematically according to their mutation rate (m: *L*ow or *H*igh), read length (len: 50 bp or 100 bp) and error rate (e: *L*ow or *H*igh). For example, mH.len100.eL refers to the profile with high mutation rate, 100 bp reads, and low error rate.

For each of the 8 profiles, we further considered the remaining two parameters each with 2 settings, leading to a total of 8×2×2=32 sets of simulated data. The first parameter is the human-mouse read ratio. We tried a low contamination rate of 9:1 (10% contamination), and a high contamination rate of 1:1 (50% contamination). The second parameter is whether reads were generated from exonic regions only, or from both exonic and non-exonic (including intronic and intergenic) regions. Since the human and mouse genomes are more similar in exonic regions, we considered the exome case to test if it would reduce read alignment and variant calling accuracies.

### Simulated data generation

We downloaded the human and mouse reference sequences hg19 and mm10, respectively, from the UCSC Table Browser [16, 17]. For each set of parameter values, we used DWGSIM ( http://sourceforge.net/apps/mediawiki/dnaa/index.php?title=Whole_Genome_Simulation) to generate human and mouse reads from the corresponding references according to the specified mutation rate, read length and error rate from the specified regions (whole genomes or exomes). Exonic regions were defined according to RefSeq [18]. The amount of data generated was equivalent to having 60 reads covering the involved regions on average. For the case with non-exonic reads, in order to perform the large number of simulation runs within a reasonable amount of time, we focused on human chromosome 14 and mouse chromosome 12, which are the two most similar chromosomes in the two genomes [19, 20], to study the worst-case situation. Reads were generated from these two chromosomes, and were allowed to be aligned to anywhere in the genomes. We have also repeated the simulations with reads generated from the whole genomes for two parameter profiles, to check how much the results would be affected by focusing on the two chromosomes.

After generating reads from each genome, we mixed them according to the specified human-mouse read ratio so that the total length of the reads is equivalent to 60 fold of the simulated regions on the human genome. This "60 × average read depth" can be considered as the amount of data perceived by someone unaware of the mouse contamination. The actual effective average read depth for the covered human regions was 60 × × 0.9 = 54 × and 60 × × 0.5 = 30 × for the human-mouse mixing ratios of 9:1 and 1:1, respectively.

### Sequence alignment and calling of genetic variants

According to the read handling strategy, we aligned the generated reads to one or both reference genomes. We used the highly accurate alignment tool Bowtie2 [15] for read alignment. In an earlier version of Bowtie2, it could not handle the artificial combined human and mouse genome. We therefore also used another popular tool, BWA [21], for the combined reference strategy, to see whether the results would be different. Since some of the required alignment results are common for the different strategies, we devised a scheme to maximize the reuse of these results. The details can be found in Additional file 1.

We then identified genetic variants from alignment results using SAMtools [22]. We considered only variants with a Phred-scale quality score [23] of 13 or above (the default value of samtools), which corresponds to an error probability of 10^-1.3^=0.05012≈5*%*.

### Evaluation metrics

The effectiveness of the three strategies was compared at three levels. First, the alignment results were compared to the actual origin of the reads to determine alignment performance. Second, SNVs called according to the alignment results were compared to the actual generated variants, to determine variant calling performance. Finally, the functional significance of the variants was evaluated by the performance of calling non-synonymous variants at genic regions. At all three levels, the performance of the three strategies were quantified by their false discovery rate (FDR) and false negative rate (FNR), defined as  and , respectively. For read alignment, TP is the number of human reads correctly aligned to the correct position in the human reference, FP is the number of human or mouse reads incorrectly aligned to the human reference, and FN is the number of human reads not aligned to the human reference. For variant calling, TP is the number of synthesized human SNVs successfully identified by the calling pipeline, FP is the number of identified SNVs not actually synthesized, and FN is the number of synthesized human SNVs missed by the calling pipeline.

As a baseline for evaluating how close the performance of the three strategies was to the best case, we also produced a set of data generated from human DNA only, and used the direct mapping strategy to align the reads. Due to the simulated mutations, sequencing errors and non-unique sequences, even in this baseline case the FDR and FNR could be non-zero.

### Nasopharyngeal carcinoma sequencing data

We further tested the three strategies using DNA sequencing data from an NPC xenograft (C15 [24]) and an NPC cell line (C666-1 [25]). We estimated the mouse contamination level in C15 to be 29.6%, based on the number of human and mouse leptin present as determined by real time PCR using a standard curve. We performed deep sequencing to produce 2.56G and 2.51G reads for C15 and C666-1, respectively, which correspond to 82.3 × and 80.9 × coverage of the human reference genome hg19.

As with the simulated data, we applied the three strategies to align sequencing reads to the human and mouse genomes, and identified genetic variants accordingly. We considered the whole human and mouse genomes instead of only human chromosome 14 and mouse chromosome 12. As the actual origin of each sequencing read and the true set of genetic variants are not known, we focused on comparing the numbers of aligned reads and variants called by the three strategies. Since C666-1 should be free of mouse contamination, the direct mapping strategy should produce the best results. We used it as a control to test if there were any drawbacks of the filtering and combined reference strategies.

## Results and discussion

### Simulated data

The complete set of results for all 32 combinations of parameter values is given in the Additional file 2. Here we first use the profile mH.len100.eL to compare the direct mapping ("Direct"), filtering ("Filtering") and combined reference ("Combined") strategies, using the dataset with no contamination ("No contamination") as control, based on Bowtie2 alignments. Afterwards we will compare the results based on different setting profiles and extend the comparison to include results based on BWA alignments. In each of these subsequent parts of analysis, we fix the values of all parameters except one, so that any difference in the alignment and variant calling accuracies must be due to this parameter chosen to have varying values.

#### Special handling is necessary in detecting functionally important SNVs

**Figure 1:**
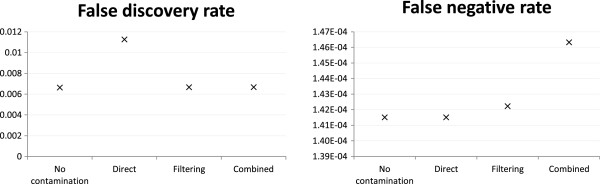
**Alignment accuracy of the base case.** Alignment accuracy of different strategies under the mH.len100.eL setting with 1:1 human-mouse mixing ratio and reads generated from both exonic and non-exonic regions of human chromosome 14 and mouse chromosome 12.

**Figure 2:**
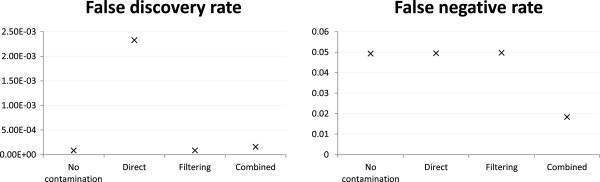
**Variant calling accuracy of the base case.** Variant calling accuracy of different strategies under the mH.len100.eL setting with 1:1 human-mouse mixing ratio and reads generated from both exonic and non-exonic regions of human chromosome 14 and mouse chromosome 12.

Figures 1 and 2 respectively show the read alignment and variant calling results based on the dataset with reads generated from both exonic and non-exonic regions of human chromosome 14 and mouse chromosome 12, and a human-mouse mixing ratio of 1:1. In both tasks, the filtering and combined reference strategies achieved lower FDRs than direct mapping. In fact, the FDRs of these two strategies were close to the best-case scenario with no contamination. This performance gain came with a price of a higher alignment FNR, especially for the combined reference strategy. Interestingly, for variant calling, the combined reference strategy actually achieved a lower FNR, probably due to a smaller amount of mouse reads wrongly aligned to the human reference that could confuse the variant caller. Nonetheless, all these differences in both FDR and FNR were within a small percentage, and thus the cost-effectiveness of the two special handling strategies was not immediately clear.

A more obvious difference between the three strategies was revealed when their ability to call non-synonymous SNVs was compared (Table 2). While the filtering strategy did not identify any false non-synonymous SNVs and the combined reference strategy identified 3, the direct mapping strategy identified 47 of them. These false positives could seriously affect experimental validations and downstream analyses. The direct mapping strategy also missed a lot more true non-synonymous SNVs than the combined reference strategy. These results show that although overall the alignment and variant calling accuracies of the filtering and combined reference strategies were not substantially better than direct mapping, they could really help in identifying the genetic variants of potential functional significance.

**Table 2:** **False non-synonymous SNVs called by the different strategies, with reads generated from both exonic and non-exonic regions**

Strategy	False positive non-synonymous SNVs	False negative non-synonymous SNVs
No contamination	0	228
Direct	47	251
Filtering	0	265
Combined	3	70

False non-synonymous SNVs called by the different strategies, and true non-synonymous SNVs missed by them, under the mH.len100.eL setting with 1:1 human-mouse mixing ratio and reads generated from both exonic and non-exonic regions of human chromosome 14 and mouse chromosome 12.

We repeated the simulations for two parameter profiles but had the reads generated from the whole genomes instead, to check if the alignment (Table S3 vs. Table S8) and variant calling (Table S9 vs. S15) accuracies would be affected. We also compared the variant calls when only variants with quality score larger than 13 were considered (Table S9) and when all identified variants were considered (Table S10). The relative FDR and FNR of the different strategies remained almost unaffected, except when the absolute differences were very small and were sensitive to small fluctuations.

#### Direct mapping is sensitive to contamination rate, while filtering is more affected by data volume

We then explored how the detection of genetic variants would be affected by the contamination level. We compared the results at human-mouse read ratios of 9:1 and 1:1. We also included a data set with the same ratio as the 1:1 set, but a doubled sequencing depth of 60 × from each genome, to study the effects of data volume. We call these three data sets "54 ×:6 ×", "30 ×:30 ×" and "60 ×:60 ×", respectively, to reflect the effective depths of reads from the human and mouse genomes.

**Figure 3:**
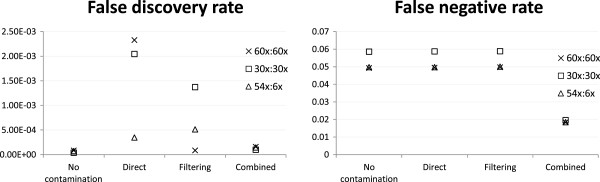
**Variant calling accuracy with various human-mouse mixing ratios.** Variant calling accuracy of different strategies under the mH.len100.eL setting with various human-mouse mixing ratios and reads generated from both exonic and non-exonic regions of human chromosome 14 and mouse chromosome 12. For the no-contamination case, only human reads of the three data sets were used.

Figure 3 shows that both direct mapping and filtering performed better with 54 ×:6 × than 30 ×:30 ×, which is expected as the former has a lower contamination level. In contrast, the performance of the combined reference strategy remained almost the same in the two cases, showing that it was not sensitive to the contamination level.

In terms of FDR, the filtering strategy worked best with 60 ×:60 ×, followed by 54 ×:6 × and worst with 30 ×:30 ×. Its performance thus appears to depend more on the actual amount of human data present (60 ×, 54 × and 30 ×, respectively) than the contamination level. For the direct mapping strategy, it performed the best with the 54 ×:6 × set, followed by 30 ×:30 × and 60 ×:60 ×, suggesting that this strategy is more affected by the contamination level (human-mouse ratio 9:1, 1:1 and 1:1, respectively). It is worth noting that even though the 60 ×:60 × set contained more reads than the 30 ×:30 × set at the same contamination level, the variants identified by the direct mapping strategy had a higher FDR from the former, which suggests that when this strategy is used, producing more reads may not help when the contamination level is high.

#### Exome sequencing is much more affected by contamination

**Figure 4:**
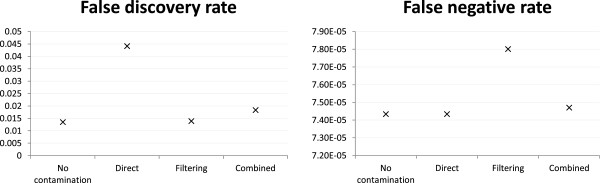
**Alignment accuracy of exonic reads.** Alignment accuracy of different strategies under the mH.len100.eL setting with 1:1 human-mouse mixing ratio and reads generated from exonic regions only of the whole human and mouse genomes.

Next we studied the effects of contamination on exome sequencing. Figure 4 shows that in terms of read alignment, the FDR of the direct mapping strategy (0.044) was substantially higher than both filtering (0.014) and combined reference (0.018). This difference is much larger than the one when reads were generated from both exonic and non-exonic regions (Figure 1, 0.011 for direct mapping vs. 0.007 for both filtering and combined reference), indicating that a lot more exonic reads were incorrectly aligned by the direct mapping strategy due to the higher sequence similarity between human and mouse exons than other genomic regions. The lower FDR of the filtering strategy came with a price of a slightly higher FNR, while the FNR for the combined reference strategy was almost the same as direct mapping.

**Figure 5:**
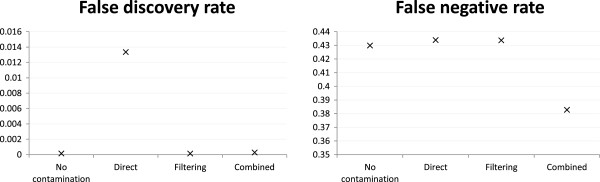
**Variant calling accuracy of exonic reads.** Variant calling accuracy of different strategies under the mH.len100.eL setting with 1:1 human-mouse mixing ratio and reads generated from exonic regions only of the whole human and mouse genomes.

For the task of calling genetic variants, the filtering and combined reference strategies were again superior to direct mapping in terms of FDR, with almost all variants they called being true positives (Figure 5). In terms of FNR, all strategies appeared to perform poorly with 38-44% true variants being missed. However, even in the no-contamination case the FNR was about 43%, showing that these variants were intrinsically difficult to call regardless of the contamination level.

For the task of identifying non-synonymous SNVs, the number of false positives detected by the direct mapping strategy was much larger than filtering and combined reference (Table 3). The absolute number is also substantially larger than when reads were generated from both exonic and non-exonic regions (Table 2). These results show that for exome sequencing, special handling is necessary for calling variants accurately.

**Table 3:** **False non-synonymous SNVs called by the different strategies, with reads generated from only exonic regions**

Strategy	False positive non-synonymous SNVs	False negative non-synonymous SNVs
No contamination	5	159,014
Direct	551	160,374
Filtering	5	159,989
Combined	16	150,988

False non-synonymous SNVs called by the different strategies, and true non-synonymous SNVs missed by them, under the mH.len100.eL setting with 1:1 human-mouse mixing ratio and reads generated from exonic regions only of human chromosome 14 and mouse chromosome 12.

#### For the combined reference strategy, Bowtie2 aligned fewer human reads correctly but resulted in more correctly called variants

All the above comparisons were based on Bowtie2 alignments. To see how much the results depend on the alignment method, we have also used BWA to align reads for a subset of the settings. Here we discuss the results based on the base case mH.len100.eL.

**Figure 6:**
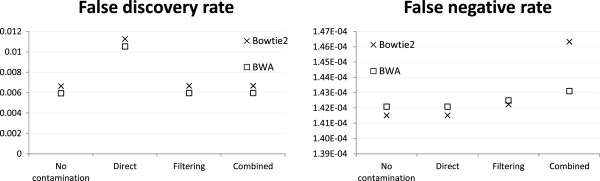
**Alignment accuracy based on two alignment methods.** Alignment accuracy of different strategies based on two alignment methods, under the mH.len100.eL setting with 1:1 human-mouse mixing ratio and reads generated from both exonic and non-exonic regions of human chromosome 14 and mouse chromosome 12.

**Figure 7:**
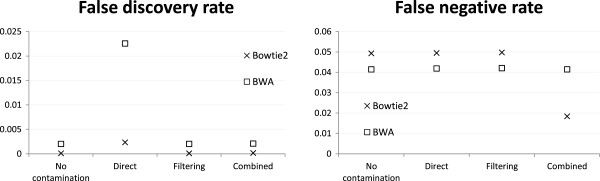
**Variant calling accuracy based on two alignment methods.** Variant calling accuracy of different strategies based on two alignment methods, under the mH.len100.eL setting with 1:1 human-mouse mixing ratio and reads generated from both exonic and non-exonic regions of human chromosome 14 and mouse chromosome 12.

In terms of read alignment, the two alignment methods produced almost the same FDR and FNR for all three strategies, except that the FNR was lower for the combined reference strategy when BWA was used (Figure 6). Surprisingly, the variant calling FNR of the combined reference strategy was higher with BWA alignment (Figure 7). This result suggests that there were some reads that Bowtie2 could not align or gave a low alignment score when the combined reference strategy was used, which were likely reads with highly similar sequences in the human and mouse genomes. While some of these were legitimate human reads, there were probably also a similar amount of mouse reads not wrongly aligned to the human reference. The net result of simultaneously having more false negative alignments and less false positive alignments seemed to have made variant calling easier and led to the lower false negative variant calls.

For the direct mapping strategy, the variant calling FDR was higher than the other two strategies, and the performance difference was more prominent with BWA alignment.

#### Effectiveness of filtering and combined reference is insensitive to sequencing error rate

**Figure 8:**
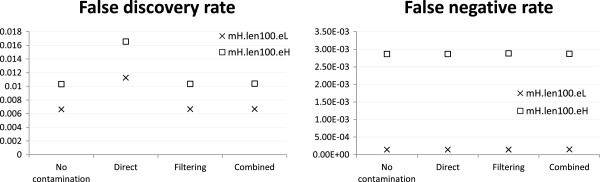
**Alignment accuracy at different sequencing error rates.** Alignment accuracy of different strategies under the mH.len100.eL and mH.len100.eH settings with 1:1 human-mouse mixing ratio and reads generated from both exonic and non-exonic regions of human chromosome 14 and mouse chromosome 12.

So far we have focused on simulation results based on the mH.len100.eL profile. We now examine how the performance of the different strategies would be affected by the rate of sequencing error. At both low and high error rates, all three strategies achieved similar FNR as the no-contamination case, and only the direct mapping strategy had a higher FDR (Figure 8). These results suggest that the filtering and combined reference strategies were equally effective in both scenarios with their results insensitive to the tested sequencing error rates.

#### Special handling has a slightly greater benefit when reads are short

**Figure 9:**
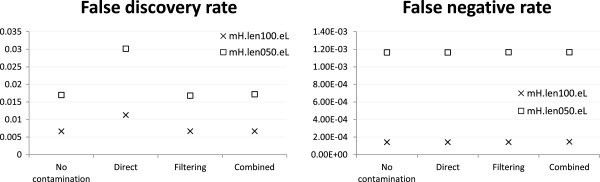
**Alignment accuracy at different read lengths.** Alignment accuracy of different strategies under the mH.len100.eL and mH.len050.eL settings with 1:1 human-mouse mixing ratio and reads generated from both exonic and non-exonic regions of human chromosome 14 and mouse chromosome 12.

Finally, we checked how the performance of the different strategies would be affected by read length. Figure 9 shows that in general all strategies achieved better performance when reads were longer, which is expected as longer reads are easier to align correctly. The filtering and combined reference strategies consistently performed better than the direct mapping strategy in terms of FDR, with a slightly larger performance difference when reads were short. Again, there were no significant differences between the three strategies in terms of FNR.

### Nasopharyngeal carcinoma sequencing data

We then applied the three alignment strategies to our NPC sequencing data. Table 4 shows the alignment statistics. For C666-1, which is expected to contain no mouse contamination, the filtering strategy aligned about 6 million fewer reads to the human reference as they were aligned to the mouse reference. Most of them should be false negatives. In comparison, the combined reference strategy aligned only about 350 thousand reads to the mouse reference, but had 60 million more reads unaligned to either reference as compared to the filtering strategy, most of which likely had high similarity to some regions in both references. Taking the number of reads aligned to the mouse reference as a fraction of the number of reads aligned to either reference, the estimated mouse contamination rates based on the direct mapping, filtering and combined reference strategies are 0.25%, 0.26% and 0.01%, respectively. These low values are all consistent with the expectation of zero contamination in the data.

**Table 4:** **Alignment statistics of the NPC data**

Mapping strategy	Reference	Mapped reads	Unmapped reads
C666-1			
Direct	hg19	2,399,396,153	111,814,507
	mm10*	6,121,700	2,505,088,960
Filtering	hg19**	2,392,763,986	111,689,642
Combined	hg19	2,340,660,573	170,203,874
	mm10	346,213	
C15			
Direct	hg19	1,786,870,566	768,689,454
	mm10*	681,133,817	1,874,426,203
Filtering	hg19**	1,768,136,467	85,616,069
Combined	hg19	1,784,069,633	95,357,988
	mm10	676,132,399	

*For comparison, we also aligned all reads to the mouse reference.

**These are the numbers of reads aligned to the human reference after discarding those aligned to the mouse reference.

For C15, when all reads were aligned to the mouse reference, about 681 million reads were successfully aligned. This number is close to the number of reads aligned to the mouse reference by the combined reference strategy (about 676 million), and thus can be used to estimate the amount of mouse contamination in the data. Again, taking it as a ratio of the total number of reads aligned to either reference, the estimated contamination rates were 27.6%, 27.8% and 27.5% for the direct mapping, filtering and combined reference strategies, respectively, which are all close to the 29.6% based on our leptin RT-PCR results. With this fairly high contamination rate, we expected special handling strategies would be necessary according to our simulation results above.

**Figure 10:**
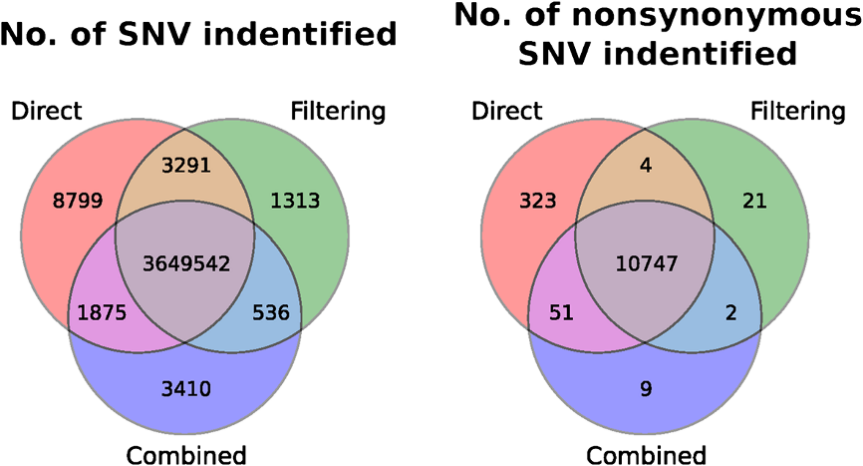
**Number of SNVs identified from C15 according to the three alignment strategies.**

Indeed, when we compared the numbers of SNVs identified by the three strategies (Figure 10, left panel), we found that while a large number of (about 3.6 million) variants were commonly found by all three strategies (this large number is expected due to a known homozygous mutation in the mismatch repair gene hPMS1 in C666-1, and the expression of high levels of the viral oncogene LMP1 in C15, which can induce genomic instability and increase the number of mutations [26]), there were also a significant number identified by only some strategies. For instance, the direct mapping strategy identified about 8,800 variants not identified by the other strategies, most of which are believed to be false positives due to the mouse contamination. In contrast, only about 1,300 variants were uniquely identified by the filtering strategy. Interestingly, there were also about 3,400 variants only identified by the combined reference strategy. In our simulation this strategy had the lowest FNR in many settings (see Figure 2 for example), therefore we believe some of these uniquely identified variants are legitimate variants that were missed by the other two strategies. Similar trends are also seen for the non-synonymous SNVs (Figure 10, right panel), where direct mapping detected 323 unique SNVs that are likely false positives, and the combined-reference strategy detected 9 potentially real SNVs missed by the other two strategies.

## Conclusions

In this paper, we have used a large number of simulations to compare the performance of the direct mapping, filtering and combined reference strategies when human sequencing reads are contaminated with mouse data. We have shown that in general, the two special handling strategies (filtering and combined reference) performed better than the direct mapping strategy that simply aligned all human and mouse reads to the human reference.

We found that in terms of calling non-synonymous SNVs, special handling methods were able to identify much fewer false positives than direct mapping, especially when reads were generated from exons only. We recommend that when the precision of the identified non-synonymous SNVs is more important than coverage, special handling should be applied. This recommendation is supported by the large number of variants only identified by direct mapping from C15, most of which are believed to be false positives.

In other situations, our simulation results provide information for evaluating the cost-effectiveness of special handling. In particular, we have shown that there are situations in which the direct mapping strategy performed only slightly worse than having special handling. One may use standard read processing pipelines to save extra bioinformatics cost in such situations.

In our simulations the combined reference strategy usually identified more true genetic variants than filtering at about the same precision. If special handling is to be applied, we suggest that the combined reference strategy could be a better choice.

Overall, the best strategy to take would depend on the particular parameter setting, project goal, sample size, and resources available. We suggest using our simulation results directly, or to perform similar simulations, to estimate the differences in alignment and variant calling accuracies of the different strategies. More fine-grained analyses could also be performed, for example to investigate these performance differences when only a certain number of the most confident variants from each strategy are considered. These results would suggest the potential numbers of false positives and false negatives in these top cases, which could guide the calculation of the relative computational and experimental costs when scaling up the number of samples.

## Electronic supplementary material

Additional file 1: **Additional material note.** Minimizing sequence alignment runs when obtaining results for all parameter setting profiles. (PDF 30 KB)

Additional file 2: **Full set of simulation results.** (PDF 9 KB)

## Abbreviations

PDX: Patient-derived tumor xenograft
SNV: Single nucleotide variant
NPC: Nasopharyngeal carcinoma
FDR: False discovery rate
FNR: False negative rate.
