# Supplementary material for: Are special read alignment strategies necessary and cost-effective when handling sequencing reads from patient-derived tumor xenografts?
Source: BMC Genomics. 2014 Dec 23;15(1):1172. doi: 10.1186/1471-2164-15-1172 (PMC4326289; doi:10.1186/1471-2164-15-1172)
Supplement: Supplementary file 1 — Additional file 1: Additional material note. Minimizing sequence alignment runs when obtaining results for all parameter setting profiles. (PDF 30 KB) [file 12864_2014_6992_MOESM1_ESM.pdf]

1 Supplementary Materials

1.1 Minimizing sequence alignment runs when obtaining results for all parameter setting profiles

In our simulation study, a large number of parameter value combinations were considered. It would take a long time if we perform sequence alignment for each combination separately. In order to minimize the number of sequence alignment runs, we developed a pipeline for maximizing the reuse of alignment results as follows.

For each combination of mutation rate, read length, error rate and sequenced region (exonic only or both exonic and non-exonic), we first produced 6 sets of basic alignment results (Table 1). They are named according to the source genome from which data (d) were generated, and the target reference (r) to which the reads were aligned. For the source genome, "H" and "M" represent the human and mouse genomes, respectively. For the target reference, "H", "M" and "H+M" represent the human, mouse, and combined reference, respectively. For example, the mapping of sequencing reads generated from the human genome to the mouse reference is named as dHrM.

Table 1: The six sets of basic alignment results.

		Sequencing Data (**d**)	
		**H**uman	**M**ouse
Reference (**r**)	**H**uman	dHrH	dMrH
	**M**ouse	dHrM	dMrM
	**H**uman+**M**ouse	dHrH+M	dMrH+M

Based on these 6 basic sets of alignment results, the results of all four strategies (direct mapping, filtering, mapping to combined reference, and control case with no contamination) were generated using set operations (Table 2). This way of producing the alignment results for the four strategies saved a substantial amount of time spent on sequence alignment by reusing the alignment results. Specifically, the alignment dHrH was used three times and dMrH was used two times. Likewise, in the tests that involved different ratios of human and mouse reads, by using down-sampling with different mixing ratios of mouse and human reads, we avoided generating sequencing reads for each setting from scratch and aligning them to reference sequences.

In our simulations involving exonic and non-exonic regions of human chromosome 14 and mouse chromosome 12, each round of alignment with 60x read depth took around 1.5 hours. If one was to consider the whole genome instead of the two selected chromosomes, the actual data size, and thus the alignment time, would be at least twenty times bigger. Reducing the number of alignments can significantly reduce simulation time.

Table 2: Obtaining alignment results for the four strategies based on the 6 basic sets.

strategy	Set operations
Direct	dHrH $\cup$ dMrH
Filtering	(dHrH $\setminus$ dHrM) $\cup$ (dMrH $\setminus$ dMrM)
Combined	dHrH+M $\cup$ dMrH+M
No contamination	dHrH

Note: The operator symbol "\" denotes the set difference operation.
