# Supplementary material for: Are special read alignment strategies necessary and cost-effective when handling sequencing reads from patient-derived tumor xenografts?
Source: BMC Genomics. 2014 Dec 23;15(1):1172. doi: 10.1186/1471-2164-15-1172 (PMC4326289; doi:10.1186/1471-2164-15-1172)
Supplement: Supplementary file 2 — Additional file 2: Full set of simulation results. (PDF 9 KB) [file 12864_2014_6992_MOESM2_ESM.pdf]

| Table | Alignment method | Coverage | Target regions | Human:mouse read depth | Evaluation level |
|---|---|---|---|---|---|
| S3 | Bowtie2 | hg19:chr14+mm10:chr12 | Exonic+non-exonic | 60x:60x | Alignment |
| S4 | Bowtie2 | Whole hg19+Whole mm10 | Exonic | 60x:60x | Alignment |
| S5 | Bowtie2 | hg19:chr14+mm10:chr12 | Exonic+non-exonic | 30x:30x | Alignment |
| S6 | Bowtie2 | hg19:chr14+mm10:chr12 | Exonic+non-exonic | 54x:6x | Alignment |
| S7 | BWA | hg19:chr14+mm10:chr12 | Exonic+non-exonic | 60x:60x | Alignment |
| S8 | Bowtie2 | Whole hg19+Whole mm10 | Exonic+non-exonic | 60x:60x | Alignment |
| S9 | Bowtie2 | hg19:chr14+mm10:chr12 | Exonic+non-exonic | 60x:60x | Variant calling (score>13) |
| S10 | Bowtie2 | hg19:chr14+mm10:chr12 | Exonic+non-exonic | 60x:60x | Variant calling (all) |
| S11 | Bowtie2 | Whole hg19+Whole mm10 | Exonic | 60x:60x | Variant calling (score>13) |
| S12 | Bowtie2 | hg19:chr14+mm10:chr12 | Exonic+non-exonic | 30x:30x | Variant calling (score>13) |
| S13 | Bowtie2 | hg19:chr14+mm10:chr12 | Exonic+non-exonic | 54x:6x | Variant calling (score>13) |
| S14 | BWA | hg19:chr14+mm10:chr12 | Exonic+non-exonic | 60x:60x | Variant calling (score>13) |
| S15 | Bowtie2 | Whole hg19+Whole mm10 | Exonic+non-exonic | 60x:60x | Variant calling (score>13) |
| S16 | Bowtie2 | hg19:chr14+mm10:chr12 | Exonic+non-exonic | 60x:60x | Non-synonymous SNVs (score>13) |
| S17 | Bowtie2 | Whole hg19+Whole mm10 | Exonic | 60x:60x | Non-synonymous SNVs (score>13) |
